# Drug interaction between a selective serotonin reuptake inhibitor and a triptan leading to serotonin toxicity: a case report and review of the literature

**DOI:** 10.1186/s13256-021-02946-8

**Published:** 2021-07-26

**Authors:** Gilbert Jin, Philip Stokes

**Affiliations:** Redcliffe Hospital, Anzac Avenue, Redcliffe, QLD 4020 Australia

**Keywords:** Serotonin toxicity, Serotonin reuptake inhibitors, Serotonin 5-HT1 receptor agonists, Triptans, Adverse effects, Drug interaction

## Abstract

**Background:**

Serotonin toxicity is a known side effect of selective serotonin reuptake inhibitors and has previously also been described as a possible side effect of 5-hydroxytryptamine receptor agonist (triptan) medications. However, the literature is conflicted about the risk of developing serotonin toxicity as a result of drug interaction between selective serotonin reuptake inhibitors and triptans.

**Case presentation:**

A 30-year-old Caucasian woman with a history of depression on regular fluvoxamine presented to the emergency department with right-sided facial and lower limb twitching. The patient had recently been prescribed sumatriptan for migraines and had taken her first ever dose shortly prior to the onset of symptoms. She was tachycardic, diaphoretic, and hypertonic on initial assessment with bilateral lower limb and ocular clonus. Electrocardiogram showed sinus tachycardia with QT interval under the treatment interval, and pathology and imaging findings were unremarkable. Her symptoms improved with supportive management and cyproheptadine.

**Conclusions:**

This patient’s presentation fulfilled both Sternbach and Hunter criteria for serotonin toxicity, illustrating a potential case of serotonin toxicity as a result of drug interaction between a selective serotonin reuptake inhibitor and a triptan.

## Background

As prescriptions of serotonergic medications have increased in recent years, serotonin toxicity has become an increasingly prevalent phenomenon [1]. Selective serotonin reuptake inhibitor (SSRI) and selective norepinephrine reuptake inhibitor (SNRI) medications are commonly prescribed for management of mood and anxiety disorders, and are frequently implicated in presentations of serotonin toxicity [2].

5-hydroxytryptamine receptor agonists, also known as serotonin receptor agonists or triptans, are commonly prescribed for the management of migraine. They are commonly coprescribed with SSRIs and SNRIs and have been previously implicated in cases of serotonin toxicity as a result of drug interaction, although such cases are rare [3].

Serotonin toxicity remains a clinical diagnosis, and can be a challenging one to make. Due to its wide spectrum of severity and easily overlooked symptoms, diagnosis can often be missed, especially if the patient does not present with a clear history of overdose [2]. It can also occur as a result of drug interaction, and should be an important differential diagnosis in any patient known to be taking serotonergic medications.

This case report aims to discuss a case of drug interaction between a triptan and an SSRI leading to a suspected case of serotonin toxicity.

## Case presentation

A 30-year-old Caucasian woman presented to the emergency department (ED) with right-sided facial and lower limb twitching. She also reported a headache for the past 5 days, similar to her previous migraines. She had taken a dose of sumatriptan approximately 6–8 hours prior to presentation to alleviate the pain. This medication had been newly prescribed by her general practitioner with no history of prior use by the patient. Shortly after taking the sumatriptan, the patient developed gradually worsening right-sided facial and lower limb twitching, with discomfort in the right arm and right leg and associated difficulty with speech. She had taken paracetamol at home prior to this event and denied ingestion of any other medications, including prescription medications as well as herbal and over-the-counter supplements. She also denied any recent alcohol or illicit drug use.

Her past medical history was significant for longstanding depression for which she was on regular fluvoxamine 100 mg once daily, as well as hemiplegic migraines (usually managed with simple analgesia), endometriosis, and paroxysmal supraventricular tachycardia. She denied any recent changes in her fluvoxamine dose. Her other regular medications included pregabalin and tranexamic acid, which she did not take on the day of presentation. She had no significant family history and denied regular alcohol or drug use. She denied any regular smoking history.

On initial assessment, she had a heart rate of 120 beats per minute and blood pressure of 144/96 mmHg, but otherwise normal vital signs and a Glasgow Coma Scale (GCS) score of 15. Temperature was normal at 36.5 °C. She had notably diaphoretic palms and a resting tremor in her right arm and leg. Initial ophthalmic assessment showed bilaterally sluggish and dilated pupils with ocular clonus. She had bilateral lower limb hypertonicity and hyperreflexia with six to seven beats of inducible clonus in both ankles. Power and sensation were normal in all four limbs, and full neurological examination was otherwise unremarkable. There were no other significant findings on physical examination.

Initial electrocardiogram (ECG) showed sinus tachycardia with a QT interval below the treatment line on QT nomogram. Full blood count, electrolytes, liver function tests, and serum beta human chorionic gonadotropin (HCG) were unremarkable. Computed tomography (CT) scan of her head showed no evidence of intracranial lesion and was grossly normal for age.

Based on her clinical presentation and history of SSRI use, the patient was diagnosed with serotonin toxicity. Differential diagnoses at the time included atypical focal seizure, alternate drug toxidrome (such as anticholinergic or sympathomimetic toxicity) or withdrawal phenomenon.

The patient was placed on telemetry and was given supportive treatment with slow intravenous fluids as well as a dose of 12 mg oral cyproheptadine, a potent antihistamine and serotonin antagonist. Her fluvoxamine and sumatriptan were withheld, and she was kept in the ED short-stay unit overnight for a prolonged period of observation. Periodic reviews throughout her admission showed an incremental improvement in her serotonergic symptoms, with improvement in her motor symptoms and agitation, as well as her ocular and lower limb clonus.

On her morning review, the patient had returned to baseline with resolution of her clonus and tremor and a completely normal repeat neurological examination, aside from some mild residual lower limb hyperreflexia. She was discharged home with instructions to withhold her fluvoxamine for 24 hours and to avoid taking any further concomitant triptan medications in the future, to be followed up by her usual general practitioner.

## Discussion and conclusions

In summary, this case describes a 30-year-old woman who presented with a likely case of serotonin toxicity as a result of drug interaction between her regular SSRI and a newly prescribed triptan medication. While there have been scattered case reports of this in the literature, very few have described a case of serotonin toxicity as a direct result of drug interaction that fulfills formal diagnostic criteria.

Serotonin toxicity is a clinical toxidrome characterized by a triad of autonomic hyperactivity, neuromuscular excitation, and altered mental state [2], and can be a potentially life-threatening side effect of serotonergic medications if left untreated [1]. It is a clinical diagnosis and can have a wide spectrum of symptoms and severity, and as such is often underrecognized. In recent years, a number of diagnostic criteria have been proposed to provide a more objective basis for diagnosis. The two most commonly used diagnostic criteria in current clinical practice are the Sternbach and Hunter criteria, as presented in Table 1 [4, 5].

**Table 1:** Hunter and Sternbach criteria for serotonin toxicity

Hunter and Sternbach criteria for serotonin toxicity	
*Hunter criteria *[4] One or more of the following in the presence of a serotonergic drug: Hypertonia Inducible clonus and agitation or diaphoresis Ocular clonus and agitation or diaphoresis Spontaneous clonus Tremor and hyperreflexia Temperature > 38 °C and ocular or inducible clonus	*Sternbach criteria* [5] Addition or increase in dose of serotonergic agent No commencement or increase in dose of neuroleptic treatment prior to onset of symptoms At least three of the following: Agitation Diaphoresis Diarrhea Fever Hyperreflexia Incoordination Myoclonus Shivering Tremor

Management of serotonin toxicity is typically supportive in nature, in addition to withdrawal of serotonergic medications, but can differ depending on the severity of presentation. In severe cases, treatment may necessitate airway and cardiorespiratory support, as well as muscle paralysis and active cooling to prevent hyperthermia and muscle rigidity. Mild-to-moderate cases often require only a period of observation and symptomatic management with benzodiazepines or cyproheptadine, and antiemetics [2].

In 2006, the United States Food and Drug Administration (FDA) issued an alert regarding a potential risk of developing serotonin toxicity as a result of concomitant use of SSRI or SNRI antidepressants with triptans [6]. Since then, the literature has been largely critical of the FDA’s position. Subsequent analyses of the case reports on which the FDA alert was based have found that a number of them did not actually meet diagnostic criteria for serotonin toxicity [3, 7]. A position paper from the American Headache Society in 2010 found that the quality of evidence supporting the FDA’s recommendation was poor and that there was insufficient evidence to support limiting the coprescription of SSRIs/SNRIs and triptans [8].

A subsequent retrospective database study of 19,017 patients who were coprescribed triptans and SSRIs/SNRIs found only 7 patients who met diagnostic criteria for serotonin toxicity, of which only 4 met both Sternbach and Hunter criteria. It concluded that the risk of developing serotonin toxicity from concomitant administration of SSRIs/SNRIs and triptans was low and recommended that the FDA advisory be reconsidered [9]. It has been postulated that the reason for this may lie in the affinity of triptan medications to certain serotonin receptor subtypes, with a higher affinity to serotonin 1B and 1D receptors. In contrast, emerging evidence suggests that serotonin syndrome is more heavily mediated by serotonin 1A or 2A receptors, for which triptans have only a low affinity [9].

However, as this case shows, while the risk of such drug interaction is low, it is not absent. The patient in this case had a likely diagnosis of serotonin toxicity as a result of drug interaction between her fluvoxamine and sumatriptan. While in ideal circumstances she would have had formal laboratory drug testing to confirm the drugs of exposure and to exclude any confounding agents, these tests are simply not realistic to perform in an emergency department environment. She did, however, satisfy clinical criteria for serotonin toxicity, which remains the mainstay of diagnosis to guide patient management. She met both Sternbach and Hunter criteria for serotonin toxicity, presenting with agitation, tremor, hyperreflexia, and both spontaneous and inducible clonus, including limb and ocular clonus. Further supporting the diagnosis were her tachycardia and mydriasis, as well as the complete resolution of her symptoms with administration of cyproheptadine, a specific histamine antagonist.

In conclusion, this case report describes a case of probable serotonin toxicity as a consequence of coadministration of an SSRI and triptan. Clinicians should be aware of the risk and counsel patients on the potential for drug interaction, and should also have a clinical index of suspicion in patients presenting with serotonergic symptoms who are on such medications, even without a history of overdose. Recognition of serotonin toxicity is key in managing these patients in an appropriate and timely fashion.

## Data Availability

Not applicable.

## Abbreviations

SSRI: Selective serotonin reuptake inhibitor
SNRI: Selective norepinephrine reuptake inhibitor
ED: Emergency department
GCS: Glasgow Coma Scale
ECG: Electrocardiogram
CT: Computed tomography
FDA: Food and Drug Administration
beta HCG: Beta human chorionic gonadotropin
